# Monitoring *Coxiella burnetii* Infection in Naturally Infected Dairy Sheep Flocks Throughout Four Lambing Seasons and Investigation of Viable Bacteria

**DOI:** 10.3389/fvets.2020.00352

**Published:** 2020-07-10

**Authors:** Raquel Álvarez-Alonso, Ion I. Zendoia, Jesús F. Barandika, Isabel Jado, Ana Hurtado, Ceferino M. López, Ana L. García-Pérez

**Affiliations:** ^1^Animal Health Department, NEIKER-Instituto Vasco de Investigación y Desarrollo Agrario, Derio, Spain; ^2^Laboratory of Special Pathogens, Instituto de Salud Carlos III, Centro Nacional de Microbiología, Madrid, Spain; ^3^Department of Animal Pathology, Veterinary Faculty, University of Santiago de Compostela, Lugo, Spain

**Keywords:** *Coxiella burnetii*, Q fever, sheep, genotypes, viability, lambing, environment

## Abstract

Progression of *Coxiella burnetii* infection in four naturally infected sheep flocks, and in their farm environment, was monitored throughout four lambing seasons. Flocks with an active infection were selected based on the presence of *C. burnetii* DNA in bulk-tank milk (BTM) and a high seroprevalence in yearlings during the previous milking period (Spring 2015). During four consecutive lambing seasons (2015/16–2018/19), samples were collected within 1 week after each lambing period from animals (vaginal swabs, milk and feces from ewes, and yearlings) and the environment (dust indoor sheep premises). BTM samples and aerosols (outdoors and indoors) were monthly collected between lambing and the end of milking. Real-time PCR analyses showed different trends in *C. burnetii* shedding in the flocks, with a general progressive decrease in bacterial shedding throughout the years, interrupted in three flocks by peaks of reinfection associated with specific management practices. A significant relationship was found between *C. burnetii* fecal shedding and the bacterial burden detected in dust, whereas shedding by vaginal route affected the detection of *C. burnetii* in indoor aerosols. Three genotypes were identified: SNP8 (three flocks, 52.9% of the samples), SNP1 (two flocks, 44.8% samples), and SNP5 (one flock, two environmental samples). *Coxiella burnetii* viability in dust measured by culture in Vero cells was demonstrated in two of the flocks, even during the fourth lambing season. The results showed that infection can remain active for over 5 years if effective control and biosafety measures are not correctly implemented.

## Introduction

Q fever is a widespread zoonosis caused by the intracellular bacterium *Coxiella burnetii*. Goats and sheep are considered the main reservoir of *C. burnetii*, and both have a significant role as source of human infection (1, 2). Q fever causes abortions in small ruminants (3), and once *C. burnetii* enters into a flock, infection spreads rapidly. Infected animals shed *C. burnetii* through birth products, vaginal fluids, feces, milk, and urine for several weeks after abortion or normal parturition (4–10), but the bacterial load shed by aborted animals is higher than that shed by those that deliver normally (9). Abortion rates due to *C. burnetii* are especially high in goats (up to 70–90%) (4, 11) but lower in sheep (usually below 6%) (12). These low rates can be considered normal by the farmer, and consequently, samples of aborted animals are not submitted for diagnostic testing. Therefore, Q fever is not diagnosed, control measures are not implemented, and the infection can be maintained in a sheep flock throughout consecutive lambings. In a recent study carried out in dairy sheep flocks, *C. burnetii* shedding through milk was still observed in several flocks 10 years after first detection (13). This suggests that flock management practices together with lack of control measures implemented can cause periodical reactivation of *C. burnetii* infection (14–17).

*Coxiella burnetii* shedding by infected animals, together with their movements in indoor animal premises, promotes the formation of contaminated aerosols. Bacterial load in aerosols is the highest at the peak of abortions (4) and also correlates with the number of shedders in the flock (18). The progression of natural infection by *C. burnetii* in sheep flocks during several breeding seasons has not been fully investigated; thus, the length of time that the infection remains active in the flock is unknown. It is known that in the breeding seasons that follow an outbreak of abortion by Q fever, abortions decrease in sheep, and *C. burnetii* shedding naturally declines (19). Vaccination with phase I vaccine helped to limit bacterial shedding in ewes and yearlings from infected flocks in the two first years, resulting in a complete clearance of the infection after 4 years of vaccine implementation (20). However, *C. burnetii* DNA was still detected in dust samples in the fourth season after vaccination (20). Presence of *Coxiella* DNA in dust collected in farm premises has been reported in several studies (4, 9, 20, 21), but the time *Coxiella* remains viable has been scarcely investigated (4, 21). Kersh et al. (21) still found viable *Coxiella* in a goat farm in the kidding season that followed an abortion outbreak. In fact, the small-cell variant (SCV) is a spore-like form of *C. burnetii*, which can survive during long periods of time in the environment (22). Results pointed out that loads of viable *C. burnetii* are at the highest level during lambing/abortion period and progressively decrease thereafter until no viable bacteria are detected 2 months after the last parturition (4).

The genotype of *C. burnetii* could affect the course of infection (23, 24). In Spain, there is not much information about genotypes of *C. burnetii* involved in Q fever cases, neither in humans nor in animals. Recent studies carried out in Northern Spain identified goats rather than sheep as the main source of Q fever for humans, with pneumonia as the main symptom (4, 25–27), but interestingly, sheep and goats share the same *C. burnetii* genotypes in this area (4, 13, 27). Therefore, further studies are needed to better understand the epidemiological features of *C. burnetii* infection in sheep. This work was aimed at studying the progress of *Coxiella* infection throughout four lambing seasons in four dairy sheep flocks in semiextensive management systems in which no vaccination program was implemented. Genotyping of the strains involved and the investigation of *C. burnetii* viability in consecutive breeding seasons would help to better understand Q fever infection in sheep.

## Materials and Methods

### Flocks Selection and Sampling Approach

*Latxa* is the dairy breed of sheep in the Basque Country. Lambing takes place once per year, between November and January for ewes and between March and April for yearlings. Animals are housed indoors in winter and at night and in rainy weather during the milking season, which ends in June–July. After that period, flocks are moved to communal mountain pastures where sheep share grazing areas with other sheep flocks, cattle, and horses, and wildlife, mainly wild boar, roe deer, badger, and foxes.

Four sheep flocks that tested real-time PCR positive to *C. burnetii* on bulk-tank milk (BTM) samples collected in March–April 2015 were selected. These flocks also showed a high seroprevalence against *C. burnetii* measured by ELISA in yearlings (Table 1). Both results suggested that *C. burnetii* infection was active in these flocks. Farmers were interested in collaborating and studying the evolution of the infection throughout four lambing seasons: 2015/2016, 2016/2017, 2017/2018, and 2018/2019. Flocks had never been vaccinated against this pathogen. It should be noted that, at the beginning of this study, there was a stock rupture in the production of the phase I inactivated vaccine, so vaccination could not be considered. A questionnaire was conducted to collect data on census, farm characteristics, management system, abortion history, and hygiene and biosecurity measures implemented in each farm. According to farmers' perception, significant abortions were not reported in the years prior to the study. However, one of them reported that a family member had suffered from pneumonia in the 2014/2015 production season, but since hospitalization was not needed, the etiological agent was not identified. For the correct management of the placentas, farmers were offered a freezer and biohazardous waste disposal containers, which, once filled, would be removed for incineration of infectious material. The goal was to quickly remove potentially infective material from the farm environment, thereby reducing possible environmental contamination. Three of the farmers performed this procedure, while the fourth (flock 2) had a type of slatted floor that resulted in placentas falling directly into the slurry pit. Table 1 summarizes information about the selected flocks. Two of the flocks (flocks 2 and 3) used to buy animals from other flocks. Flock 4 had old animal premises and moved to a new farm in the lambing season 2018/2019.

**Table 1:** General information of the sheep flocks included in the study.

	**Flock 1**	**Flock 2**	**Flock 3**	**Flock 4**
**CENSUS**				
Ewes	416	250	543	411
Yearlings	80	50	87	83
Communal pastures	Ewes	Ewes and yearlings	Ewes	Ewes and yearlings
Cattle in the farm	Yes	No	No	No
Goats in the farm	Yes	No	No	No
Purchase of animals	No	Yes	Yes	No
Abortions >3%	No	No	No	No
**CHARACTERISTICS OF THE SHEEP PREMISES**				
Year of construction	1995	2009	2007	1975^*^
Ventilation	Regular	Bad	Regular	Bad
Slatted floor	Yes	Yes	No	No
Straw bedding	Yearlings	No	Yes	Yes
Water source	Well	Well	Tap water	Tap water
Frequency of manure removal	Daily	1/year	2/year	2/year
**BIOSECURITY MEASURES**				
Management of placentas	Cremation	Manure	Cremation	Cremation
Exclusive cloth	No	No	No	No
Access of visits	Yes	Yes	Yes	Yes
Other measures	No	No	No	No
**Q FEVER STATUS (2014/15)**				
Seroprevalence (%)				
Ewes	26.7	40.0	13.3	26.7
Yearlings	66.7	46.7	46.7	53.3
BTM ELISA	Positive	Positive	Positive	Positive
BTM PCR	Positive	Positive	Positive	Positive
**RANGE OF ANIMALS ANALYZED PER LAMBING SEASON**				
Ewes^†^	30–40 (110)	30–61^§^ (131)	30–68^§^ (138)	30–40 (110)
Yearlings	28–40 (132)	7–28 (62)	7–28 (71)	30–40 (140)

* *New sheep premises for the period 2018/19*.

† *Ewes were not sampled in the 2018–2019 lambing season*.

§ *Purchased ewes included*.

Flocks were visited at lambing during four consecutive lambing seasons. During the first three seasons, samples were collected from ewes and yearlings and purchased animals (if any). During the last lambing season, only yearlings were sampled. Vaginal exudates (collected with swabs without medium), milk, and feces were taken from a maximum of 40 ewes and/or 40 yearlings within 1 week after parturition to evaluate *C. burnetii* shedding (Table 1). During these visits, environmental samples consisting of duplicates of 8–10 dust samples were taken from different surfaces of the animal premises to detect the presence of *C. burnetii* DNA and for further viability studies (one duplicate kept at −80°C). In addition, monthly visits were made until the end of the milking period, and a BTM sample was collected to monitor *C. burnetii* shedding at the flock level, as well as aerosols collected indoor and outdoor sheep premises. The air sampler “MD8” Sartorius (Goettingen, Germany) was used, performing an aspiration of 100 L/min air for 10 min. The air passed through a gelatin filter adapted to the equipment, which was analyzed in the laboratory by real-time PCR in order to detect the presence of *C. burnetii* DNA in the aerosols generated at the farm.

### Molecular Analyses

#### DNA Extraction and Real-Time PCR

DNA was extracted using the QIAmp DNA Blood Mini Kit (Qiagen Hilden, Germany) with some modifications. Briefly, milk or feces were mixed with 180 μl of ATL buffer (Qiagen, Hilden, Germany) and digested with 20 μl of proteinase K (8 mg/ml) for 30 min at 70°C before DNA extraction. Vaginal or dust swabs were treated with 300 μl of TE buffer (10 mM Tris base, 1 mM EDTA, pH 8) before being mixed with ATL and proteinase K for 1 h at 56°C. The initial treatment of the gelatin filters from the air sampler was done as previously described (18). Negative extraction controls were included every 10 samples to rule out DNA contamination. The presence of *C. burnetii* DNA was investigated by a real-time PCR procedure targeting the transposon-like repetitive region *IS1111* of *C. burnetii* genome (28). A commercial internal amplification control (IAC) (TaqMan® Exogenous Internal Positive Control, Thermo Fisher Scientific) was included in the assay to monitor for PCR inhibitors.

#### Genotyping

A selection of animal and environmental samples positive by real-time PCR with Ct <31 were genotyped using a previously described single-nucleotide polymorphism (SNP) genotyping assay that detects 10 discriminatory SNPs by real-time PCR (29). SNPs were identified and selected by Huijsmans et al. (29) on the basis of both the consensus sequence generated from 100,000 bp of the five known whole genome sequences of *C. burnetii* (RSA493, RSA331, CbuG_Q212, Cbuk_Q154, and 5J108 111 Dugway) and an *in silico* investigation of their discriminatory power using BLAST (29). SNPs 769, 2287, 4439, 4557, 4844, 5423, and 6025 (positions indicated in the reference sequence RSA493, GenBank accession no. AE016828.2) are located within single-copy genes. SNPs 7087, 7726, and 7974 are located within the multicopy insertion sequence IS*1111* (positions within the first IS*1111* encountered, as indicated in the strain RSA493 reference sequence, GenBank accession no. AE016828.2), which is distributed throughout the *C. burnetii* genome. Ten real-time PCR reactions were performed per sample, each including two primers and two MGB® TaqMan probes to detect point mutations. Each 20 μl PCR mixture contained 625 nM of each primer, 125 nM of each probe, 1 × Taq Mix ABsolute (Thermo Fisher Scientific), and 5 μl of template DNA. PCR reactions were run on a BioRad platform (CFX96™ RTi-PCR Detection System) using the following program: 15 min at 95°C, and 45 cycles of 3 s at 95°C, and 30 s at 60°C.

A selection of samples were also genotyped by multispacer sequence typing (MST) of eight spacers (Cox2, Cox5, Cox18, Cox22, Cox37, Cox51, Cox56, and Cox61) as previously described (30), with small modifications. Briefly, two four-plex PCR reactions were carried out followed by individual amplifications for each spacer region. Each amplicon was then purified and sequenced, and genotypes were identified by comparison with the database at https://ifr48.timone.univ-mrs.fr/mst/coxiella_burnetii/.

### Serological Analyses: Enzyme-Linked Immunosorbent Assay

In order to evaluate seroprevalence against *C. burnetii*, individual milk samples were centrifuged, and milk sera were tested for Q fever antibodies using an ELISA test (LSIVET Ruminant Serum/Milk Q Fever kit; Thermo Fisher Scientific). An index (S/P) of the tested milk serum optical density to optical density of the positive control ratio was calculated according to the manufacturer's instructions. Individual milk samples with S/P indices ≤ 0.4 were considered negative, while samples with S/P >0.4 were considered positive.

### Viability Studies

#### Ethics

Experimental studies were carried out in BSL3 facilities and consisted of experimental inoculations in 6-week-old BALB/c male mice combined with cell culture. Permission was obtained from the Ethical & Animal Welfare Committee (Bizkaiko Foru Aldundia, document 3/2017 v02, Reg. 32243 25 June 2018).

#### Isolation

Environmental viability of *C. burnetii* was assessed on dust samples collected after yearlings lambing under the assumption that dust deposited at that time would had been originated from aerosols generated during both ewes and yearlings lambing. Viability studies of C. *burnetii* in environmental samples were carried out by passage through mice and culture in Vero cells [African green monkey epithelial cells VERO C1008 (Vero 76, clone E6, Vero E6 ATCC® CRL-1586™)]. Dust samples were homogenized and prepared as detailed elsewhere (4). The quantification of *C. burnetii* genome equivalents (GE) in each homogenate was carried out by quantitative real-time PCR (qPCR) using 5 μl of DNA (in triplicates) and specific primers and a probe targeting the *IS1111* gene as described elsewhere (28). In each qPCR run, a standard curve was generated using 10-fold serial dilutions of a known concentration of Nine Mile (RSA439) phase II strain of *C. burnetii* DNA. After quantification, aliquots of 200–500 μl were prepared from each dust homogenate, containing approximately 10^2^-10^3^ *C. burnetii* GE. These aliquots were inoculated intraperitoneally in four mice each; a dust homogenate with viable *C. burnetii* (4) collected from a goat farm in 2017 and stored at −80°C was used as a positive control. As determined in previous studies (4, 31), mice were euthanized on days +14 and +21 p.i., and spleens were removed. The level of splenomegaly was determined from the ratio of the spleen weight to body weight. Half of the spleen from each mouse was processed for DNA extraction and real-time PCR amplification as fully detailed elsewhere (4). Positive samples were subjected to qPCR to quantify the number of *C. burnetii* GE detected in spleen in order to compare it with the number of GE inoculated; when the number of *C. burnetii* GE recovered from the spleen was equal or higher than the GE inoculated, *C. burnetii* was considered to have multiplied *in vivo*.

For qPCR-positive samples, the second half of the spleen was homogenized with 700 μl Dulbecco's modified Eagle's medium (DMEM) medium and 2% fetal bovine serum (FBS) in a TissueLyser. A hundred microliters of each homogenate were placed on shell vials (SV) containing Vero cells, as fully detailed elsewhere (4). Briefly, after harvesting *C. burnetii* from SV on day 6 p.i., three passages of 1,000 μl of harvested cells were transferred at weekly intervals into T25 culture flasks containing a Vero layer. At day 6 p.i. and before each passage, 200 μl was collected for DNA extraction and qPCR, following procedures described above. Cultures that maintained *C. burnetii* growth during the second or third passage were considered to be positive. Uninfected control cells were kept close to infected cells to rule out possible cross-contaminations.

### Statistical Analyses

The possible influence of the different factors studied, i.e., flock (categorical; flock 1, flock 2, flock 3, flock 4), period of lambing (categorical; ewes/yearlings), and lambing season (categorical; 2015–2016, 2016–2017, 2017–2018, 2018–2019) over positive or negative *C. burnetii* animals shedding through vaginal fluids, feces, or milk was analyzed using a logistic regression. The final model was selected as the one with the lowest Akaike's information criterion (AIC) value from all of the models performed. Odds ratio values were computed by raising “e” to the power of the logistic coefficient over the reference category.

Cohen kappa statistics were used for assessing agreement between shedding by different excretion routes and ELISA results. The symmetry of disagreement between them was evaluated with McNemar's chi square test.

The risk of environmental (dust, indoor/outdoor aerosol) contamination by *C. burnetii* was evaluated with a data mining classification tree using “rpart” package (32); dust, indoor aerosols, and outdoor aerosols were continuous dependent variables (expressed in Ct values in real-time PCR). Classification and regression tree (CRT) identifies variables that divide environmental (dust/aerosols) results into homogeneous subgroups with distinct patterns of *C. burnetii* contamination. The CRT model provides a way to identify main factors. CRT evaluates all the values of the potential factor using, as a criterion, the significance of a statistical ANOVA test and split maximizing the between-groups sum of squares, selecting the best predictor variable to form the branch in a classification tree; successively splitting in data set makes increasingly homogeneous nodes in relation to the dependent variable. This process continues until the classification tree is fully grown. Figures for CRT were performed by “rpart.plot” package (33).

The degree of splenomegaly in experimental mice was evaluated with a Welch two sample *t* test to correct homoscedasticity. Linear regression was performed with the purpose of analyzing any relationship between *C. burnetii* GE load present in the inoculum injected to mice and the GE of *C. burnetii* recovered from the spleen of experimental animals. Log-transformed data were used in both analyses. All statistical analyses were performed using the statistical software R version 3.6.2 (34).

## Results

### *Coxiella burnetii* Shedding and Serological Response in Ewes and Yearlings

**Figure 1:**
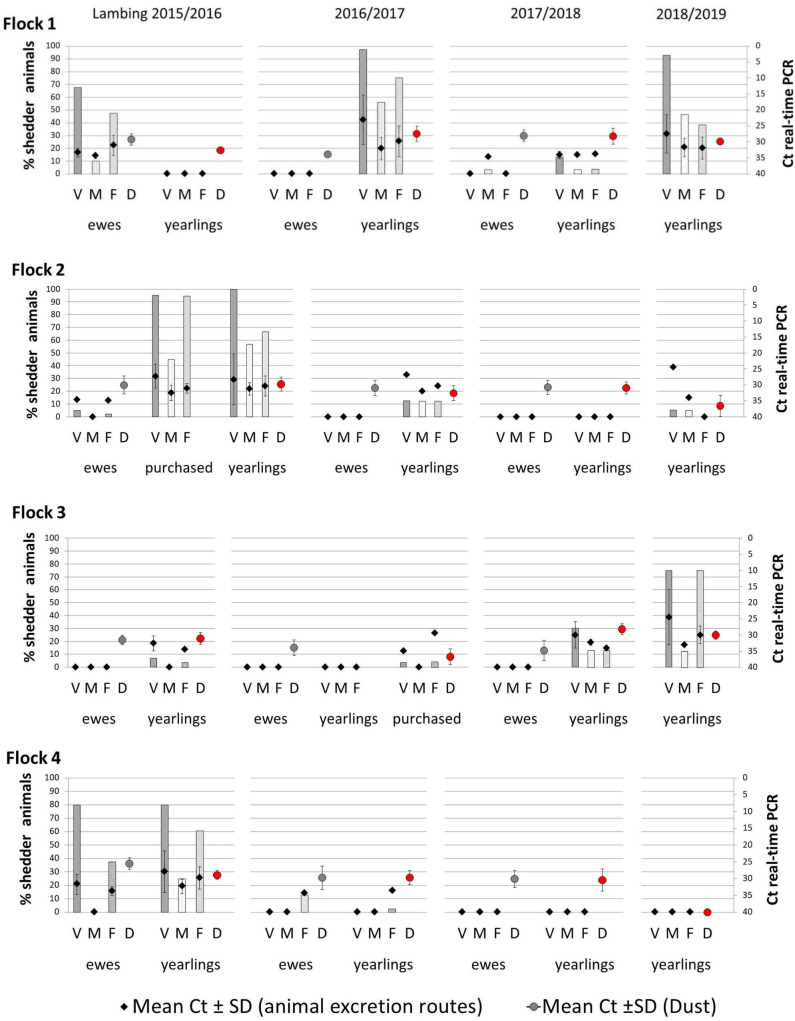
Percentage of *Coxiella burnetii* shedders (ewes, yearlings, and, if applicable, purchased animals) throughout the four lambing seasons in the four studied flocks, through vaginal fluids (V), milk (M), and feces (F) with mean Ct values ± SD, represented with diamonds. The circles represent the mean of the Ct values ± SD obtained in real-time PCR from dust samples taken during ewes lambings (gray circles) and yearlings lambings (red circles). Ewes were not sampled in the fourth lambing season and are not represented.

Real-time PCR results showed different trends in *Coxiella* shedding throughout the 4-year study in the studied flocks (Figure 1). Overall, the percentage of *C. burnetii* shedders was significantly higher in the first lambing season (2015/2016) (Table 2). In the first lambing season, flock 1 showed a high-moderate percentage of ewes shedding *C. burnetii* by different routes (vaginal fluids > feces > milk), but no shedders were detected in the group of yearlings. The following lambing seasons (2016/2017 and 2017/2018) ewes barely shed *C. burnetii*, whereas yearlings showed a reactivation of the infection in the second and fourth lambing seasons. The situation in flock 2 was affected by the reported purchase of a group of 30 pregnant ewes in December 2015. These animals became infected when they entered into the contaminated animal premises and shed high *C. burnetii* loads at lambing. This caused the reactivation of infection in the flock, which mainly affected the yearlings while the proportion of ewe shedders of that season was low (Figure 1). Afterwards, in the following seasons, flock 2 showed a decrease in the percentage of animal shedders. Patterns of infection in flock 3 were the opposite, with a low proportion or absence of *C. burnetii* shedders during the first two lambing seasons and reactivation of infection in yearlings in the last two seasons. Flock 3 introduced a new group of pregnant ewes (*N* = 62) on the 20th March 2017, 1 week before the lambing of yearlings. Later on, purchased animals showed to be infected at lambing (7% animal shedders, 2/28, in samplings carried out between 27th March and 19th April). Flock 4 showed a high percentage of shedders during the first lambing season, in both ewes and yearlings, but a significant decrease occurred in the following lambings and no shedders were detected in the last two seasons (Figure 1). Overall, flock 4 had a significantly higher number of animal shedders than flocks 2 and 3, but lower than flock 1 (Table 2).

**Table 2:** Logistic regression model for the prevalence of shedders.

	**Estimate**	***Z* value**	**Pr (**>|**t**|**)**	**OR**	**95% CI**
Intercept	0.4718	2.233	0.0255	1.60	1.06–2.43
**Flock 4 (Ref.)**					
Flock 1	0.6048	2.816	0.0050	1.83	1.20–2.80
Flock 2	−1.1090	−3.775	0.0001	0.33	0.18–0.58
Flock 3	−1.7177	−5.553	0.0001	0.18	0.10–0.32
**Yearlings (Ref.)**					
Ewes	−0.7806	−3.762	0.0001	0.46	0.30–0.68
Purchased	1.2847	4.353	0.0001	6.20	2.74–14.26
**Lambing.season 2015–2016 (Ref.)**					
Lambing.season 2016–2017	−1.7405	−7.708	0.0001	0.18	0.11–0.27
Lambing.season 2017–2018	−2.4730	−8.327	0.0001	0.08	0.05–0.15
Lambing.season 2018–2019	−0.6371	−2.166	0.0303	0.52	0.30–0.93

Shedders were more frequently found among yearlings than among ewes, but to a lesser extent than among newly introduced animals (purchased group) (Table 2). Regarding *C. burnetii* shedding loads expressed as Ct values in real-time PCR, the highest excretion levels (lowest Ct) were detected in yearlings, especially through vaginal fluids (Figure 1).

**Figure 2:**
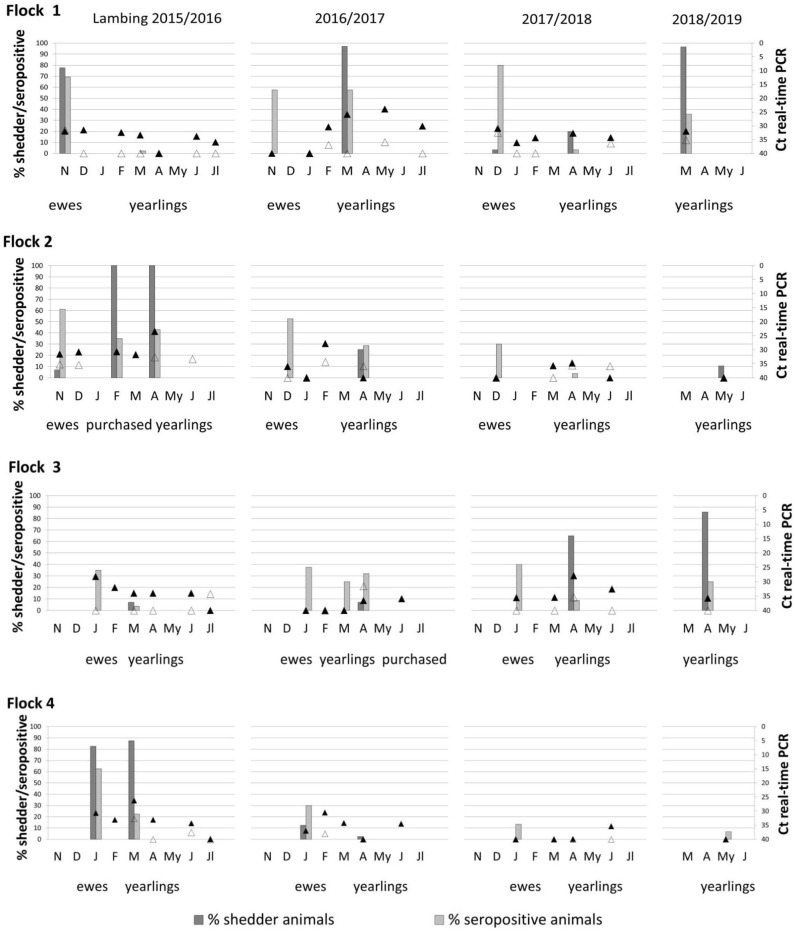
Percentage of shedder animals by at least one excretion route (vaginal fluids, feces, or milk) and seroprevalence observed corresponding to ewes and yearlings of the four studied flocks during four lambing season (bars). *Coxiella burnetii* DNA present in aerosols taken outdoor (empty triangles) and indoor (black triangles) animal premises from lambing to the end of milking season are also represented. Ewes were not sampled in the fourth lambing season and are not represented.

Comparison of seroprevalence in ewes throughout the lambing seasons showed marked differences between flocks (Figure 2). Thus, in flock 1, seroprevalence in ewes ranged between 58 and 80%; in flock 2, between 30 and 60%; in flock 3, 35–40%; and in flock 4, 13–63%. Thirty-two percent of the recently purchased ewes (9/28) in flock 3 had antibodies against *C. burnetii*, suggesting that ewes were already infected when introduced into the flock.

In yearlings, seroprevalence increased or decreased according to the trends of *C. burnetii* infection in each flock, showing in general lower seroprevalences than ewes. Flocks 2 and 4 showed a progressive decrease in seroprevalence during the study period (Figure 2). Independently of the shedding route (vaginal, feces, or milk), a high percentage of *C. burnetii* shedders was not always positively correlated with seroprevalence (Figure 2). Cohen kappa (kappa = 0.142) and McNemar's test (McNemar's chi-squared = 10.75, *df* = 1, *P* = 0.001) statistics showed a poor relationship between *C. burnetii* shedding and ELISA results. Results of *C. burnetii* shedding as well as detection of antibodies in ewes and yearlings per flock and lambing season are compiled in Table S1.

**Figure 3:**
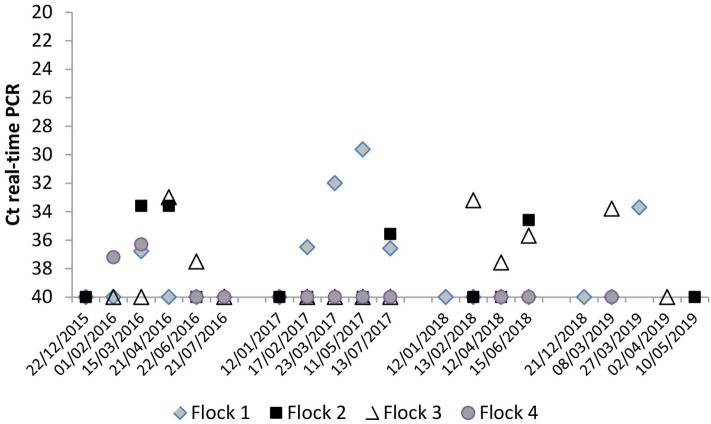
Evolution of *C. burnetii* shedding through milk measured by bulk-tank milk (BTM) real-time PCR analyses throughout the four milking periods.

The evolution of *C. burnetii* DNA presence in BTM samples during the four milking seasons is shown in Figure 3. In the first lambing season, bacterial shedding was very low in the four herds, always with Ct >30. In all milking seasons, bacterial load in BTM was higher after yearlings were included in milking (from February onwards). The highest bacterial load (Ct = 30) was found in flock 1 in the second lambing season. The reactivation of *C. burnetii* infection observed in yearlings from flocks 1 and 3 (see above) was also reflected in the bacterial load detected in BTM samples (Figure 3 and Table S1).

### *Coxiella burnetii* in Environmental Samples

**Figure 4:**
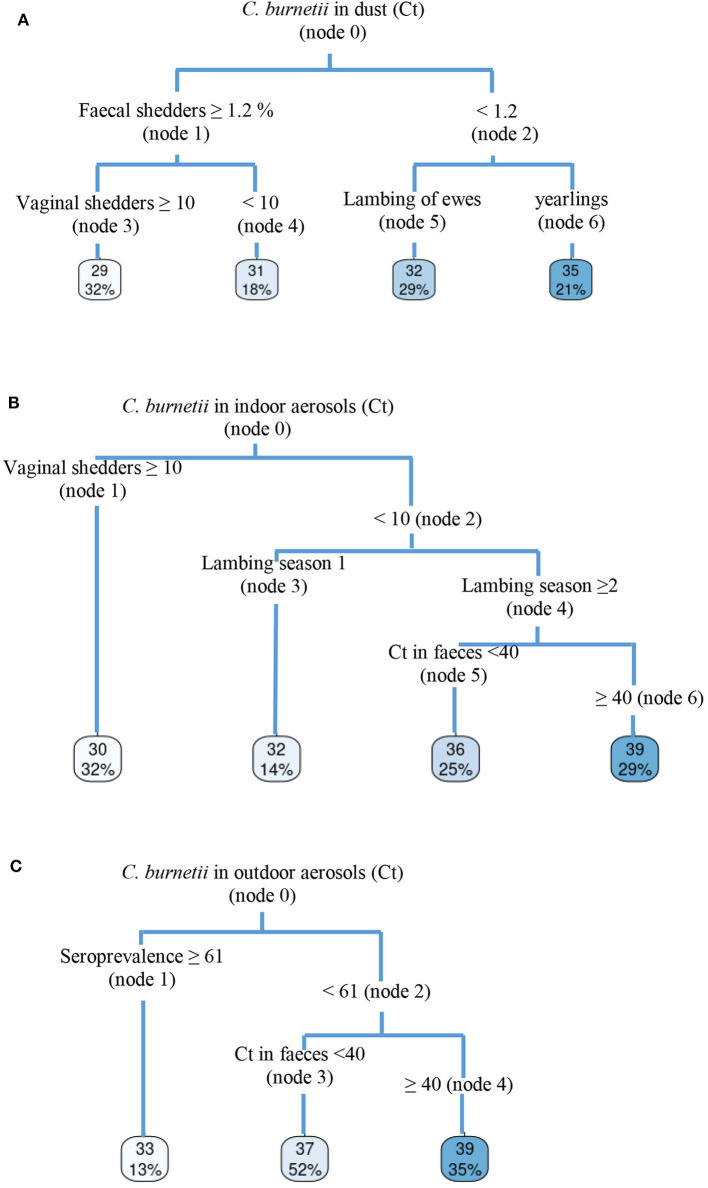
Classification and regression tree (CRT) showing determining risk-factors for *C. burnetii* detection in **(A)** dust, **(B)** indoor aerosols, and **(C)** outdoor aerosols.

Dust samples taken from different surfaces on animal premises during ewe and yearling lambings were *C. burnetii* DNA-positive throughout the 4 years of the study (ranges of Ct of 27–35), the only exception being flock 4, which became negative in the fourth lambing season. This flock had moved to new animal premises before lambing season 2018/2019 started, and *C. burnetii* DNA was not detected in dust thereafter. In the other three flocks, sporadic increases in dust bacterial load with respect to previous years were associated to increases in the number of shedders (Figure 1). The CRT algorithm stratified variables that played an important role in the amount of *C. burnetii* in dust (Figure 4A) and identified two determining factors for higher amounts of *C. burnetii* in dust (Ct = 29, 32% samplings), i.e., a percentage of fecal shedders higher than 1.2% (node 1), followed by the presence in the flock of more than 10% of shedders by vaginal route (node 3). When the presence of fecal shedders was low (node 2), the presence of *C. burnetii* in dust was determined by the lambing of ewes rather than yearlings (Figure 4A).

Results for the aerosols taken monthly indoor and outdoor of the farm from lambing until the end of the milking period are shown in Figure 2. Flocks 2 and 4 showed a progressive decrease in indoor environmental contamination during the course of the study, and in the fourth season, aerosols taken at yearling lambing were negative. On the contrary, flocks 1 and 3 showed sporadic increases in bacterial loads in aerosols due to reinfections in the flocks, and in the last lambing season, positive aerosols were still detected in the farm. The presence of *C. burnetii* in indoor aerosols was determined by the percentage of vaginal shedders (node 1) (Figure 4B), and when the percentage of shedders was below 10% (node 2), lambing season was identified as a determining factor (node 3); in the first lambing season, mean Ct value in indoor aerosols was lower than in the following seasons. From the second lambing period onwards, Ct values were determined by *C. burnetii* load shed through feces (node 5). Contamination of outdoor aerosols was less frequent, and when positive, the bacterial load was always lower (Ct > 32) than in aerosols taken indoors (Figure 2). Based on CRT algorithm results, rates of seroprevalence higher than 61% (node 1) determined the presence of positive aerosols outdoors (Figure 4C), and when seroprevalence in the flock was lower, a higher excretion of *C. burnetii* through feces (Ct <40) (node 3) was the determining factor. Raw data of real-time PCR results obtained from dust and indoors/outdoors aerosols can be found in Table S1.

### Genotypes of *C. burnetii*

A selection of samples (vaginal fluids, feces, milk, dust, and aerosols), with low Ct values in real-time PCR, was analyzed by SNP genotyping. The number of genotyped samples in each flock depended on the infection status shown in the course of the study. Thus, a total of 87 DNA samples could be genotyped (flock 1, 27 samples; flock 2, 25 samples; flock 3, 18 samples; flock 4, 17 samples), mainly from animals (61%) and dust (28%), and to a lesser extent, aerosols (11%) (Table S2). In flocks 1 and 4, only one genotype was identified (SNP8 in flock 1 and SNP1 in flock 4). In flock 2, three genotypes were identified (SNP1, SNP5, and SNP8), but SNP1 was the predominant genotype. In flock 3, two genotypes were identified (SNP1 and SNP8), but SNP8 was the predominant in both animal and environmental samples (Table 3). Overall, SNP8 was the most widespread genotype, found in three flocks and accounting for 52.9% of the samples; SNP1 was found in two flocks and 44.8% of the samples. For a selection of DNA samples (*N* = 4), the MST genotype was also determined. Two SNP1 samples from flocks 2 and 4 belonged to the MST13 genotype, while two SNP8 samples from flocks 1 and 2 corresponded with MST18. Unfortunately, no complete MST results were obtained from the only sample (dust) with SNP5 genotype.

**Table 3:** Single-nucleotide polymorphism (SNP) genotyping results from selected DNA samples obtained in each flock from animals and the environment (dust and aerosols) during the four lambing seasons.

**Lambing season**	**Origin of samples**	**Flock 1**	**Flock 2**			**Flock 3**		**Flock 4**
		**SNP8**	**SNP1**	**SNP5**	**SNP8**	**SNP1**	**SNP8**	**SNP1**
2015–2016	Ewes/yearlings^*^	5	13	–	1	1	–	12
	Dust	–	3	–	–	–	3	3
	Aerosols	–	1	1	–	–	1	1
2016–2017	Ewes/yearlings	6	1	–	–	–	1	–
	Dust	2	1	1	–	–	1	1
	Aerosols	3	1	–	–	–	–	–
2017–2018	Ewes/yearlings	–	–	–	–	–	2	–
	Dust	2	–	–	1	–	2	–
	Aerosols	1	–	–	–	–	1	–
2018–2019	Ewes/yearlings	6	1	–	–	–	4	–
	Dust	2	–	–	–	–	2	–
	Aerosols	–	–	–	–	–	–	–

* *Vaginal fluids/milk/feces*.

### Viability of *C. burnetii* in Dust

The duplicates of dust samples collected at each yearling lambing were pooled and processed to study *C. burnetii* viability using cell line culture. These accounted for 15 dust homogenates corresponding to the four lambings in flocks 1, 2, and 3, and three lambings in flock 4 (Table 4). Ct values of dust homogenates in real-time PCR ranged between 28.7 and 35.5 (Table 4). Prior to cell culture assays, each homogenate was inoculated into four 6-week-old male BALB/c mice. *C. burnetii* DNA was detected in the spleen of 20 of the 60 inoculated mice at +14 and +21 days p.i.; yet, in 3 of them, recovered GEs were below inoculated GEs. The presence of *C. burnetii* DNA was also confirmed in the four mice inoculated with the positive control (one mouse with lower GE than inoculated).

**Table 4:** Investigation of viable *C. burnetii* in dust collected at yearling lambings in the studied flocks throughout the study using Balb/c mice and culture in Vero cell lines.

			**BALB/c mice inoculation**					**Culture in cell lines (Vero E6) (GE/ml)**					
**Flock**	**Lambing period**	**Ct real-time PCR (mean)**	**No. of GE^a^ injected**	**No. of mice sacrificed at day +14 p.i.^b^**	**No. of mice sacrificed at day +21p.i**.	**No. of positive mice (day p.i.)**	**No. of GE in spleen**	**Inoculated**	**Day 6 p.i**.	**1st passage**	**2nd passage**	**3rd passage**	**Viable *C. burnetii***
1	2015–2016	32.9	3.8 × 10^2^	2	2	2 (+14)	1.7 × 10^3^	1.00 × 10^2^	0.0	0.0	0.0	0.0	No
							1.0 × 10^3^	1.00 × 10^2^	0.0	0.0	0.0	0.0	No
						1 (+21)	1.3 × 10^4^	1.00 × 10^2^	0.0	0.0	0.0	0.0	No
	2016–2017	28.8	5.8 × 10^3^	2	2	2 (+14)	3.4 × 10^4^	1.54 × 10^2^	3.44 × 10^2^	0.0	0.0	0.0	No
							1.2 × 10^5^	1.42 × 10^3^	3.71 × 10^3^	5.80 × 10^2^	0.0	0.0	No
						2 (+21)	1.1 × 10^3^	1.00 × 10^2^	2.01 × 10^3^	0.0	0.0	0.0	No
							9.8 × 10^6^	9.50 × 10^5^	8.16 × 10^5^	1.43 × 10^6^	2.88 × 10^5^	2.26 × 10^5^	Yes
	2017–2018	29.6	3.4 × 10^3^	2	2	2 (+14)	2.5 × 10^2^	1.00 × 10^2^	0.0	0.0	0.0	0.0	No
							4.1 × 10^2^	1.00 × 10^2^	0.0	0.0	0.0	0.0	No
	2018–2019	28.7	6.2 × 10^3^	2	2	2 (+14)	7.0 × 10^5^	1.20 × 10^4^	1.11 × 10^4^	9.10 × 10^3^	0.0	0.0	No
							4.5 × 10^7^	9.45 × 10^5^	4.23 × 10^5^	1.94 × 10^5^	2.22 × 10^5^	0.0	Yes
						2 (+21)	2.7 × 10^6^	3.82 × 10^6^	2.18 × 10^6^	4.70 × 10^5^	5.24 × 10^5^	1.00 × 10^5^	Yes
							5.5 × 10^6^	9.49 × 10^5^	2.76 × 10^6^	3.65 × 10^5^	3.65 × 10^5^	0.0	Yes
2	2015–2016	29.8	2.9 × 10^3^	2	2	0	0						
	2016–2017	31.9	7.5 × 10^2^	2	2	1 (+21)	8.8 × 10^3^	2.52 × 10^3^	2.10 × 10^3^	0.0	0.0	0.0	No
	2017–2018	34.2	1.7 × 10^2^	2	2	0	0						
	2018–2019	34.7	1.2 × 10^2^	2	2	0	0						
3	2015–2016	30.5	2.0 × 10^3^	2	2	1 (+14)	8.5 × 10^4^	1.00 × 10^2^	0.0	0.0	0.0	0.0	No
						2 (+21)	7.1 × 10^5^	4.51 × 10^3^	9.86 × 10^3^	1.89 × 10^3^	0.0	0.0	No
							1.2 × 10^4^	5.19 × 10^3^	1.42 × 10^4^	1.85 × 10^4^	0.0	0.0	No
	2016–2017	35.5	9.4 × 10^1^	2	2	0	0						
	2017–2018	32.9	3.9 × 10^2^	2	2	0	0						
	2018–2019	29.4	3.9 × 10^3^	2	2	1 (+14)	7.1 × 10^7^	4.45 × 10^6^	1.11 × 10^6^	4.63 × 10^5^	4.46 × 10^5^	1.00 × 10^5^	Yes
						2 (+21)	1.5 × 10^7^	3.79 × 10^6^	1.25 × 10^6^	1.99 × 10^6^	3.30 × 10^6^	1.32 × 10^6^	Yes
							5.4 × 10^7^	9.03 × 10^6^	1.95 × 10^7^	1.19 × 10^7^	6.92 × 10^6^	6.03 × 10^6^	Yes
4	2015–2016	31.9	7.4 × 10^2^	2	2	0	0						
	2016–2017	30.8	1.6 × 10^3^	2	2	0	0						
	2017–2018	33.2	3.2 × 10^2^	2	2	0	0						
Posit. Control (goats)	2017	20.9	10.7 × 10^5^	2	2	2 (+14)	3.0 × 10^7^	1.82 × 10^4^	4.88 × 10^4^	5.33 × 10^4^	8.53 × 10^4^	0.0	Yes
							9.1 × 10^6^	2.88 × 10^6^	6.90 × 10^5^	8.37 × 10^5^	2.76 × 10^5^	6.91 × 10^5^	Yes
						2 (+21)	4.8 × 10^6^	2.30 × 10^4^	2.05 × 10^4^	2.86 × 10^4^	0.0	0.0	No
							5.0 × 10^4^	3.00 × 10^2^	0.0	0.0	0.0	0.0	No

a *GE, genome equivalents of C. burnetii determined by quantitative real-time PCR targeting IS1111*.

b *p.i., postinoculation*.

Comparisons among flocks suggested that viable *C. burnetii* were more widespread in flock 1, since 13 of the 16 mice spleens were positive after inoculation with dust collected in the four lambing seasons. In flock 2, only one mouse inoculated with a dust sample taken in the second lambing season was real-time PCR positive. In flock 3, six mice inoculated with dust collected in the first and fourth lambing seasons were positive in real-time PCR. Finally, mice inoculated with dust homogenates from flock 4 were all negative.

A significant correlation was observed between the *C. burnetii* GE inoculated and the *C. burnetii* GE recovered from spleen (adjusted *R*^2^ = 0.4398, *P* = 0.0042). To assess splenomegaly, the ratio spleen of weight/live weight of mice was compared between positive mice (*C. burnetii* DNA detected in spleen by real-time PCR) and negative mice. Significant differences were observed (*t* = −3.6449; *df* = 29.512; *p* = 0.0010), and positive mice showed a ratio 1.53 times greater than negative mice.

Cultures in Vero cells of homogenates of spleens from the 20 real-time PCR-positive mice, as described above, resulted in *C. burnetii* growth in 7 mice (Table 4). Growth for at least two passages was only observed in those cases when shell vials were inoculated with at least 9.45 × 10^5^ GE. *C. burnetii* isolates were successfully cultured from dust collected in flock 1 in the second and fourth lambing season, whereas in flock 3, viable *C. burnetii* was only recovered in dust collected in the fourth lambing seasons.

SNP genotypes were analyzed in positive spleens, and SNP8 was identified in mice inoculated with dust samples from flocks 1 and 3, whereas SNP1 was identified in the only mouse positive from flock 2. Interestingly, SNP5 detected in two environmental samples in flock 2 was not recovered from mice.

## Discussion

This study shows the patterns of *C. burnetii* infection in four naturally infected dairy sheep flocks throughout four lambing seasons. The presence of animal shedders in the previous milking season and high seroprevalence in yearlings suggested at the beginning of the study that infection was active in all flocks (1, 16). It is known that shedding of *C. burnetii* by vaginal fluids, feces, and milk can persist in the breeding season that follows infection onset in small ruminant farms, even when vaccination has been implemented (20). In this study, the low percentage or even the absence of shedders among ewes (flocks 2 and 3) and yearlings (flock 1) during the first lambing season supported the suspicion that *C. burnetii* infection had been present in these flocks before the 2014/2015 season. Besides, infection was still active in some flocks in the fourth lambing season (flocks 1 and 3), leading one to think that *C. burnetii* infection can remain in a flock for more than 5 years, probably due to periodical reinfections. In fact, a previous study hypothesized that infection could be maintained for 10 years (13). The outcome of infection showed the most desirable progression in flock 4, which started with the highest percentage of animal shedders and the highest bacterial shedding in ewes and yearlings during the first lambing season, followed by a significant decrease in the following years until the infection disappeared in the fourth lambing season. Movement of the flock to new uncontaminated farm premises before lambing season 2018/2019 undoubtedly helped to keep the flock free of infection.

The fact that the bacterial load shed throughout the 4 years of the study was lower in ewes than in yearlings also supports the idea that infection had established in the flocks some time before the study started. Had the infection onset occurred before, ewes would have had time to develop immunity, whereas yearlings would have been more susceptible to the infection (9).

This study also highlighted the risk of introducing naive animals into an infected flock, which, as shown in flock 2, can cause a reactivation of the infection. Purchasing infected animals also poses a risk when introduced into a negative flock (35, 36). This was probably the situation in flock 3, where purchased pregnant ewes could have been the source of a new infection. After lambings started, 1 week after purchase, the presence of shedders was detected and moderate seroprevalence was recorded, suggesting that those animals could have been infected previously and be the source of the infection observed in yearlings during the third and fourth lambing seasons.

The four flocks were managed under a semiextensive system where animals are housed during lambing and milking and, afterwards, graze on communal mountain pastures in contact with livestock and wildlife. In a region like the Basque Country, where Q fever is endemic and vaccination is not frequently implemented, this system poses a risk for infection and reinfection (14, 15, 17). Nevertheless, the infection peak observed in yearlings in flock 1 during the second and fourth lambing seasons is difficult to explain since yearlings did not share grazing pastures with other flocks. Roe deer frequented yearling grazing fields; however, their role as infection source was ruled out after testing *Coxiella* negative by PCR analysis of their feces (data not shown). However, 5.1% of the roe deer analyzed in the same region harbored *C. burnetii* DNA (37). Therefore, the role of roe deer and other wildlife species as source of *C. burnetii* infection as reported elsewhere (38) cannot be ignored. Besides, this was the only farm that also holds beef cattle and goats as potential source for yearling infection that unfortunately could not be tested. However, cattle and goats in the farm were managed under a year-round extensive system and did not share pastures with sheep, thus minimizing opportunities for sheep cross-infection. In any case, inappropriate implementation of biosafety measures is a risk factor for flock infections that cannot be ruled out (1, 16, 36).

The interpretation of the humoral immune response against *C. burnetii* is complex and has little value at individual level because a percentage of infected animals (25–50%) do not seroconvert (6, 39–41). In this study, a commercial ELISA kit was used to measure *C. burnetii* antibodies in milk samples, since a good correlation between the level of antibodies detected in individual milk with those present in blood serum has been reported (42, 43). The marked differences in seroprevalence observed in ewes and yearlings throughout the four lambing seasons among flocks were probably associated to the exposure of animals to different loads of viable bacteria. However, as seen in this study and others (16), seroprevalence is not correlated with bacterial shedding.

The abovementioned factors, such as animal purchase or grazing in communal pastures, can favor not only *C. burnetii* infection but also the probability of infection with more than one genotype. The presence of several *Coxiella* genotypes in a farm has been previously described (9, 44–46). Here, only one genotype was detected in flocks 1 and 4, whereas in flocks 2 and 3, three and two different genotypes were detected, respectively. Interestingly, flocks with more than one genotype were those that had purchased animals. Regardless of the flock, in all cases, the more frequently found genotypes were SNP1/MST13 and SNP8/MST18. Both genotypes had been found in the region in sheep flocks (13) or in Q fever outbreaks of caprine origin associated with pneumonia in humans (4, 27). In Europe, SNP1 and SNP8 genotypes have also been associated to Q fever outbreaks (29, 45), and SNP5, only detected here in two environmental samples (one dust, one aerosol) from flock 2, has been found in goats in Belgium and France (29, 45). Regarding any possible association between genotype and infection pattern, it is noteworthy that SNP1/MST13 was the predominant genotype in flocks where infection progressed toward a gradual decrease (flocks 2 and 4), whereas genotype SNP8/MST18 predominated in the two flocks where infection reactivated in yearlings (flocks 1 and 3). However, the effect of purchase of animals on that reactivation hampers any further conclusions. Similarly, since abortion rates were very low, no associations can be inferred between pathogenicity and genotype.

Inhalation of aerosols contaminated with *C. burnetii* is the main infection route in humans (23). Wind can easily spread *Coxiella* resistance forms when climatic conditions are favorable, and therefore, environmental contamination of the surroundings of infected farms is a hotspot of concern that has been addressed by many studies (4, 9, 21, 47, 48). In this study, *Coxiella* loads were higher in aerosols taken indoors than in those taken outdoors, and levels progressively decreased during the weeks that followed the lambing seasons. These results are in agreement with those found in similar studies (4, 18, 21, 47, 48). Interestingly, detection of *C. burnetii* in indoor aerosols was dependent on the proportion of animal shedders through vaginal fluids, as reported elsewhere (18). *C. burnetii* excretion by vaginal fluids is normally higher compared to feces or milk (9), especially when infection is recent in the flock. This study also pointed out that a high seroprevalence in the flock could be an indication of a recent infection by Q fever with a higher risk of detecting contaminated aerosols by *C. burnetii* outdoors.

*Coxiella* accumulates in the dust of infected farms, and its DNA remains in dust for long periods (20, 21). Here, levels of *C. burnetii* in dust seemed to depend mainly on the number of fecal shedders, and to a lesser extent of shedders by vaginal exudates. Nevertheless, the bacterial loads detected in dust samples were low in all four sheep flocks (ranges of Ct, 27–35) compared to the loads found in other goat and sheep flocks (4, 21, 49), but similar to the Ct values obtained in some farms from the Netherlands during the large Q fever outbreak (47, 48). The fact that, in the study herein, farmers rapidly discharged the placentas contributed to reduce environmental contamination at the sheep premises. Even though the inoculation procedure in mice was carried out at the same time and using the same conditions for all samples, inoculation of dust homogenates collected during the first lambing season of yearlings in flocks 2 and 4 did not produce positive results. Inoculated GE loads (2.9 × 10^3^ in flock 2 and 7.43 × 10^2^ in flock 4) were similar to those used with samples collected from the other two flocks that multiplied in mice tissues. Interestingly, the genotype identified in these samples was SNP1, which might suggests that multiplication of low concentrations of this particular genotype is not enough to grow in mice tissues.

Despite these low contamination loads in dust, isolation of *C. burnetii* was obtained from dust collected in the second and fourth lambing seasons in flock 1 and the fourth lambing season in flock 3. These results suggest that environmental contamination on the premises and surrounding areas of farms where *C. burnetii* infection has been present for several years and the number of shedders is low is markedly lower than at farms suffering a Q fever outbreak (4). However, despite the low loads of C*. burnetii* in dust, infection risk was still present since viable *C. burnetii* were detected during the fourth lambing season in two of the studied sheep flocks.

In conclusion, this study provided valuable epidemiological data on *C. burnetii* infection in sheep and opened new questions that require further investigation. The results obtained demonstrated that if *C. burnetii* infection is not controlled using a combination of vaccination and implementation of adequate biosafety and managing procedures, an active infection and continuous shedding of viable bacteria can persist in sheep flocks for over 5 years.

## Data Availability Statement

The datasets presented in this study can be found in online repositories. The names of the repository/repositories and accession number(s) can be found in the article/Supplementary Material.

## Ethics Statement

The animal study was reviewed and approved by Ethical & Animal Welfare Committee (Bizkaiko Foru Aldundia, document 3/2017 v02, Reg. 32243 25 June 2018).

## Author Contributions

JB and AG-P: conceptualization. JB, IJ, and AG-P: methodology. CL: statistical analysis. RÁ-A, IJ, IZ, JB, and AG-P: investigation. AG-P and IJ: resources. RÁ-A and JB: data curation. RÁ-A: writing—original draft preparation. AH and AG-P: writing—review and editing. JB, AH, and AG-P: supervision. AG-P: project administration and funding acquisition. All authors contributed to manuscript revision and have read and approved the final manuscript.

## Conflict of Interest

The authors declare that the research was conducted in the absence of any commercial or financial relationships that could be construed as a potential conflict of interest.

## Supplementary Material

Table S1 Compilation of results of *Coxiella burnetii* shedding and detection of antibodies in ewes and yearlings per flock and lambing season; real-time PCR results obtained from BTM samples and from dust and indoors/outdoors aerosols.

Table S2 Results of SNP genotyping obtained from animal and environmental samples.
